# A proposal for systematic monitoring of the commercial determinants of health: a pilot study assessing the feasibility of monitoring lobbying and political donations in Australia

**DOI:** 10.1186/s12992-022-00900-x

**Published:** 2023-01-07

**Authors:** Jennifer Lacy-Nichols, Katherine Cullerton

**Affiliations:** 1 Melbourne School of Population and Global Health, Centre for Health Policy, The University of Melbourne, Level 5, 207 Bouverie St, Melbourne, VIC 3010 Australia; 2 School of Public Health, The University of Queensland, Brisbane, Australia

**Keywords:** Commercial Determinants of Health, Monitoring, Lobbying, Political donations, Corporate political activity, Data

## Abstract

**Background:**

The commercial determinants of health include a range of practices to promote business interests, often at the expense of public health. Corporate political practices, such as lobbying and campaign donations, are used to influence policy makers and foster a political and regulatory environment conducive to business interests. Despite recognition of their public health importance, thus far there are relatively few efforts to systematically monitor commercial political practices.

**Methods:**

A pilot study was conducted to explore the feasibility of systematically monitoring two political practices – lobbying and political contributions – for ‘harmful industries’ (alcohol, gambling, ultra-processed food and tobacco industries) in Australia. Potential data sources were reviewed to compare data availability and detail. Two publicly available datasets were selected for the pilot: ministerial diaries for New South Wales and annual donor filings from the Australian Electoral Commission. Google Data Studio was used to analyse and visualise findings.

**Results:**

The pilot study resulted in the creation of several interactive charts and dashboards that supported analysis and interrogation of the data. These charts helped to easily convey the volume of lobbying and political donations, as well as changes over time. For example, we found that between July 2014 and December 2020, NSW ministers had 20,607 meetings, of which 634 meetings were with harmful industries. And between 1998 and 2020, a total of $576,519,472 disclosed donations were made to political parties and other entities, of which $35,823,937 were from harmful industries.

**Conclusions:**

Opportunities to develop a program to monitor commercial political practices face several challenges including access barriers arising from poor availability and detail of data, technical barriers arising from the format of data disclosures and coding challenges arising from the diverse nature of the commercial sector. Despite these challenges, our pilot study demonstrates the potential to implement a monitoring program and to expand its scope to other commercial determinants of health.

## Introduction

Powerful commercial actors often act against the interests of public health. These activities include marketing harmful products such as tobacco and alcohol, disputing public health evidence, polluting communities, engaging in unsafe or insecure employment practices, and blocking public health policies [1, 2]. Collectively, these are referred to as the Commercial Determinants of Health (CDoH), which the World Health Organization define as the conditions, actions and omissions by corporate actors that affect health [3]. This approach is broad, recognising that commercial actors influence health in both positive and negative ways, often simultaneously.

Recognising the influence of the CDoH more broadly, there is a need for public health surveillance of commercial entities [4, 5]. One of the earlier CDoH models refers to corporations as “vectors of disease,” an analogy that, if we follow its epidemiological logic, leads us to ask why we don’t monitor corporations and other commercial entities with the same vigilance that we attend to other disease vectors [6]. Several researchers and organisations, including WHO, have called for systematic monitoring of the CDoH [7, 8]. There are many potential elements of a CDoH monitoring program. We focus here on one dimension: corporate political practices.

Powerful corporations engage in a range of political practices to influence the political environment in their favour. Researchers of the CDoH analyse these practices, providing insights on how businesses engage in lobbying, political contributions, litigation, the revolving door, issue framing, manufacturing doubt and more [7, 9–13]. Collectively, these political practices delay, block and undermine the development and implementation of the most effective and equitable policies to protect and improve public health [14]. Public health researchers have proposed different frameworks to guide data collection and analysis, including the Corporate Political Activity Framework, [15] the Policy Dystopia Model, [12] the Corporate Permeation Index, [16] the Corporate Financial Influence Index, [14] and the Commercial Determinants of Health Index [7].

Yet efforts to monitor corporate political activities face a myriad of challenges, including the most common challenge, inaccessible and incomplete data [17, 18]. Companies rarely disclose their political practices willingly. Government disclosures of their engagement with commercial entities depend on regulatory requirements, which differ dramatically around the world. Rarely is this as complete or as detailed as we would like, although Canada and Ireland offer examples of more robust lobbyist registers [19, 20]. Non-government organisations (NGOs) such as Open Secrets in the US and Transparency International’s Open Access in the EU have developed databases to monitor and analyse commercial political practices, [21] though these are limited by the data made available by governments and not widely replicated around the world. Additional insights into commercial political practices come from the process of litigation, where internal company documents are made public. A repository of evidence, beginning with tobacco industry documents, [22] has now expanded to include sugar, [23] food, [24] opioids, [25] chemicals [26], and fossil fuel industries [27]. These discovery documents offer an insider view of not just the political activities of businesses, but their intentions and strategies – information that would usually be confidential. While this is very useful data, it does not cover the day-to-day political engagement activities of corporations, such as routine lobbying. The end result, is that public health researchers are often faced with a patchy dataset (where one exists at all) or data that is so voluminous and variegated as to be unmanageable [28].

To address the problem of patchy or voluminous (unhelpful) datasets we set out to examine the possibility of systematically monitoring two political practices, lobbying and political contributions. We use the following definition of lobbying: Any direct or indirect communication with a public official that is made, managed or directed with the purpose of influencing public decision-making [29]. Political contributions include commercial donations to election campaigns, politicians, political parties as well as fundraising committees (noting that these specific categories will differ between political systems) [30]. We note that lobbying activities are not inherently problematic. Indeed, the OECD and transparency organisations recognise that lobbying is a normal and legitimate activity undertaken in democracies [29, 31]. Political science scholarship has often conceptualised lobbying as a form of mutually beneficial information exchange, recognising that public servants can benefit from the insights and expertise of different stakeholders [32]. However, there is also evidence that business interests have disproportionate access to political decision-makers compared to health advocates, raising concerns that they are able to co-opt or realign policy by overpowering other voices [33–35]. From a public health standpoint, if commercial actors whose products and practices harm health have the ear of governments, this is a public health risk. However, in the absence of systematic monitoring of corporate political practices, we cannot understand whether there is an imbalance in access.

While our interest is on measuring the practice of lobbying and making political donations, it is important to note the influence of the political context in analysing and interpreting corporate political activities. One branch of lobbying scholarship considers how political contexts influence the demand for lobbyists – arguing that governments and policy agendas can act as a ‘catalyst’ for the creation or change in behaviour of lobbyists [36, 37]. Thus, as different issues ascend or descend policy agendas, this will incentivise or disincentive some groups to lobby. Institutional and procedural contexts also shape the channels and frequency of lobbying. For example, in the European Commission, advisory committees are an important avenue for influencing policy agendas, and in the US, the Office of Management and Budget is highly influential [38, 39]. At a more macro level, a country’s political and legal systems also influence the opportunities available for corporations to influence governments, with Western liberal democracies’ (such as Australia) inclusive relationships with businesses facilitating access and influence [40]. The rules around lobbying and political donations similarly influence the nature of political activities pursued as well as the accessibility of data. While a review of the Australian lobbyist and political donation regulations is outside the scope of this paper, we direct readers to recent reviews on this subject [41–44].

With these issues in mind, we undertook a pilot study to explore the feasibility of monitoring lobbying and political donations. Lobbying and political contributions can be undertaken by actors from any sector (including public health organisations, civil society and individuals). In this paper we focus on the political activities of four harmful industries: tobacco, alcohol, gambling, and ultra-processed foods, whose products and practices have been linked to health harms [5]. In the following sections we discuss the methods and initial findings of our pilot project. We conclude with a discussion of the challenges and opportunities to develop a national program of systematic monitoring of commercial political practices. Our overarching conclusion is that truly systematic, rigorous monitoring is formidably challenging, but not impossible.

## Methods

In 2020, the Victorian Health Promotion Foundation (hereafter VicHealth) in consultation with policy, practitioner and academic stakeholders, commenced the development of a program to understand the impact of harmful industries on health and wellbeing outcomes in Australia. As a first step, VicHealth is piloting a model to systematically collate, analyse and document information on harmful industry activities in Australia, with a primary focus on the alcohol, gambling and ultra-processed food sectors, with some inclusion of tobacco. The first author (JLN) was employed to help scope, design and implement the pilot.

In consultation with an advisory group of public health researchers and NGOs, eight priority domains were selected for the pilot: lobbying, political contributions, revolving door, astroturf organisations, digital marketing, influencers, community sponsorship and corporate health promotion. Our methods for piloting a tool to monitor lobbying and political contributions are detailed here.

### Scoping

Our first step was to determine the availability of information available. For each political activity (lobbying and political donations), relevant datasets were identified and explored. The websites of the federal, state and territory governments were searched to identify records of ministerial diaries, lobbyist registers and political donation returns. While most jurisdictions provided lobbyist registers and records of political donations, only three states disclosed ministerial diaries. Table 1 documents the availability of datasets for each jurisdiction (updated 16/09/2021).

**Table 1:** Availability of data on lobbying and political contributions in Australia

Data source	Jurisdiction	Available	Information	Format	Time period	Disclosure frequency	Release date	No. docs
**Ministerial diaries**	Federal	N						
	ACT	Y	meeting date; attendees; purpose	.pdf	Jan 2018 – Jun 2021	Quarterly	ND^a^	113
	NSW	Y	meeting date; attendees; purpose	.pdf	Jul 2014 – Jun 2021	Quarterly	ND	644
	NT	N						
	QLD	Y	meeting date; attendees; purpose	.pdf	Jan 2013 – Jul 2021	Monthly	Last day of following month	1851
	SA	N						
	TAS	N						
	VIC	N						
	WA	N						
**Lobbyist register**	Federal	Y	Organisation profile^b^; clients; registered lobbyists (specific government position & date of separation)	.csv				299^c^
	ACT	Y	Organisation profile; clients; registered lobbyists (specific government position & date of separation)	webpage				52
	NSW	Y	Organisation profile; clients; registered lobbyists	webpage				157
	NT							
	QLD	Y	Organisation profile; clients; registered lobbyists (Y/N previous government representative); meeting record^d^	webpage	2013—2021			121
	SA	Y	Organisation profile; clients; registered lobbyists (specific government position & date of separation); meeting record	.csv	2018—2021			99
	TAS	Y	Organisation profile; clients; registered lobbyists	webpage				68
	VIC	Y	Organisation profile; clients; registered lobbyists (level of government position)					145
	WA	Y	Organisation profile; clients; registered lobbyists	webpage				118
**Political donation returns**	Federal	Y	Financial Year; Donor; Recipient; Date of Donation; Amount	.csv	1998—2020		Feb	1^e^
	ACT	Y	[variable] donor; address; recipient; type; amount; financial year	Webpage	1993–2021			47
	NSW	Y	[variable] Donor; Recipient; Date of Donation; Amount; purpose; type	Webpage; pdf;.csv	1999–2021			> 57,000
	NT	Y	[variable] donor; address; recipient; amount; type; date	pdf (image); webpage	2005–2021			63
	QLD	Y	Donor; recipient; date; amount; description	.csv, webpage	2013–2021			1/13,508
	SA	Y	Donor; electorate; address; recipient; date; amount	.csv	2015–2021			1
	TAS	N						
	VIC	Y	Donor; Suburb; State; Recipient; Recipient electorate; Date of Donation; type; Amount	.csv	2018–2021			1
	WA	Y	[variable] donor; address; amount; type; financial year	pdf (image)	1997–2020			432

^a^ND = not disclosed

^b^Organisation profile includes name, address and ABN

^c^‘No. docs’ for lobbyist registers refers to the number of registered lobbying firms; total documents have not yet been scoped

^d^Meeting records include: client, government representative, date of meeting and meeting purpose

^e^‘No. docs’ for political donation returns refers to the number of distinct webpages hosting information

### Data collection

Two datasets were selected for the pilot: NSW ministerial diaries and the federal record of political donation returns.

Ministerial diaries from July 2014 – December 2020 (*n* = 598) were downloaded from the NSW government website (https://www.dpc.nsw.gov.au/publications/ministers-diary-disclosures/). Adobe Acrobat DC was used to convert PDF files to.xlsx (excel spreadsheet) files. Each file was manually cleaned and formatted in Excel (e.g., removed headers and footers, formatted columns, ensured dates were consistently formatted). The final table had 20,608 rows, each representing a unique meeting.

Political contribution data (including federal and state donations) was downloaded from the Australian Electoral Commission (AEC) Transparency Register (https://transparency.aec.gov.au/AnnualDonor) as a single.csv file and imported into Excel. For missing dates, original donor returns were reviewed. For returns that listed only the month (and not the specific date), then the first of the month was used for the date. For donations with no date listed (e.g., Star Entertainment $800 donation to Lib-NSW 2018–2019), the last day of the financial year was used, (e.g., 30/06/2019). In some cases, donations were presented as an annual aggregate of several donations (e.g., Motor Trades Electoral Action Committee 1998–1999 return). In this case, the date of the last donation was used. The original returns of eight donations listed as ‘zero’ dollars were all reviewed. The original donations were for amounts less than one dollar, and all made to the Citizens Electoral Council of Australia party. These were left as $0.00 to be consistent with AEC reporting.

### Analysis

Once we had collected all the data, our next step was to organise the data so that it could be analysed and visualised. To analyse the data, the lobbying and political contribution datasets were organised into fact tables (containing the data observations, e.g., a specific political donation) and dimension tables (which contain descriptive attributes about the variables in the fact table, e.g., the political party affiliation of the donation recipient). This approach follows Tidy Data principles, [45] which aims to make ‘messy’ datasets ‘easy to manipulate, model and visualise’ by applying a similar structure. This segmentation is important for data warehouse design, which allows you to build relationships between the fact and dimension tables, so that, for example, it is possible to filter political donations by the industry affiliation of the donor. While a data warehouse was not within the scope of the pilot, it is a logical next step, thus we applied data warehouse design principles to the organisation of our data [46].

**Fig. 1:**
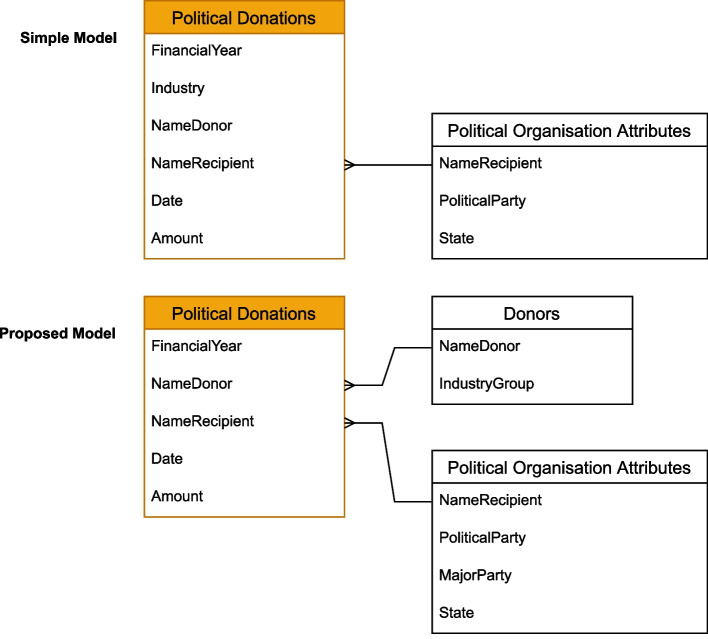
Data organisation models for political donations

For this project, two fact tables (one of ministerial meetings and a second of political donations) and four-dimension tables were created for coding purposes. The dimension tables corresponded to three variables in the fact tables: 1) harmful industry groups (alcohol, tobacco, gambling and ultra-processed foods) used to code the donors and meeting attendees; 2) ministerial portfolios used to code individual ministers; and 3) political parties used to code donor recipients. Figure 1 models the relationships between the fact tables (in orange) and the dimension tables for political contributions data.

To build the dimension tables, all values for each relevant variable (for instance a list of every donor) were copied from the fact table into a new table and duplicates were removed (case sensitive). Each unique value in the dimension table was subsequently assigned relevant attributes as detailed below.

### Harmful industry groups

For the lobbying fact table, there were 13,731 unique groups of meeting attendees, and for the political donation fact table, there were 5718 unique donors. The dimension tables coded each of these actors to a specific industry group (or ‘other’). Two different coding approaches were trialled for the lobbying and political contribution dimension tables.

### Lobbying

For this group, we focused on differentiating between health and commercial organisations. Meeting attendees (ranging from a single attendee to more than ten) were reviewed and coded to either: 1) one of the ‘harmful industries’ (tobacco, alcohol, gambling, food & drink), 2) ‘health advocacy’ or 3) ‘other’ (meetings that did not have at least one actor from these groups). Some meetings included multiple actors representing multiple industry interests. If only one of the four ‘harmful industry’ actors was present, the meeting was coded to that industry. Where multiple ‘harmful industry’ actors were present, we prioritised the industry that best aligned with the meeting purpose. Where that information was not available or insufficient, we prioritised the ‘harmful industry’ with the greatest representation in that meeting.

### Political donations

For the political contribution group, we focused on commercial attributes. All donors were first coded to their harmful industry group (or other). For those entities, we then classified them as either company or trade association and whether they were large (> = 0.5% market share), small (< 0.5% market share) or single establishments (e.g., local hotels) (using data from Euromonitor). Some commercial actors could be coded to multiple industries (e.g., Coca-Cola Amatil is both ultra-processed food and alcohol and Australian Hotels Association is both alcohol and gambling). For all commercial actors, a primary industry designation was established in consultation with colleagues at VicHealth and external experts, though we discuss challenges with this approach in the discussion.

### Ministerial portfolios

There were 43 unique ministers and 88 unique portfolios. Records of ministers’ start and end dates were accessed from the NSW government website. To facilitate analysis and enable comparisons across portfolios, 16 thematic groups of similar portfolios (e.g., health and mental health) were developed.

### Political parties and organisations

As of June 2021, Australia had 49 registered political parties (not including 25 state-affiliated parties). There were two main parties represented in the House of Representatives – the Australian Labor Party and a coalition between the Liberal Party of Australia and the Nationals – with five other parties also represented (Australian Greens, One Nation, Katter's Australia Party, Palmer United Party and Centre Alliance). The 1914 unique donor recipients were coded as one of eight major parties (Liberal, Labor, National, Greens, One Nation, Katter's Australia Party, Palmer United Party or Centre Alliance), ‘Minor Party’ or ‘Other’ (e.g., other parties or political organisations) [47]. Each recipient was also coded to its state branch (e.g., AU-QLD), or if it was a federal branch or unspecified parties they were coded to ‘Australia’. All other recipients not linked to a political party were also coded to ‘Australia’ (e.g., 250 Club Limited).

Google Data Studio was used to explore data visualisation. However, Google Data Studio had limitations in the number of dimension tables that can be linked to a fact table, thus a modified approach was used to organise and analyse the data to fit within the requirements of Google Data Studio. Figure 1 shows the simple model of political donations used in the pilot and an expanded model allowing for more relationships between tables.

Google Data Studio was used to chart and visualise the lobbying and political contribution data (and is the source for figures in the results). The lobbying and political contribution data and dimension tables were uploaded to Google sheets. Data Studio allows you to ‘blend’ two tables using one or more ‘join keys,’ to link the fact and dimension tables. The fact table can then be visualised and filtered by the different variables in the dimension table (for example, comparing the alcohol and gambling industries’ meeting with politicians, or comparing the political contributions from companies versus trade associations). For the lobbying and political contribution datasets, a series of different interactive charts were developed. These were subsequently embedded in a website developed to showcase potential outputs and tested with the advisory group. Examples of these interactive charts are included in the results.

## Results

Our pilot project resulted in the creation of two Google Data Studio reports comprising different dashboards to visualise and interact with the data we collected on lobbying and political donations. The below sections detail a sample of our preliminary empirical findings arising from these reports. These are intended to illustrate some of the insights possible from using visualisation software, as well as the value in developing a program of work to systematically monitor corporate political practices. We also hope that they highlight opportunities for future research to build on and expand this pilot, and we reflect on our learnings in the discussion.

### Lobbying

**Fig. 2:**
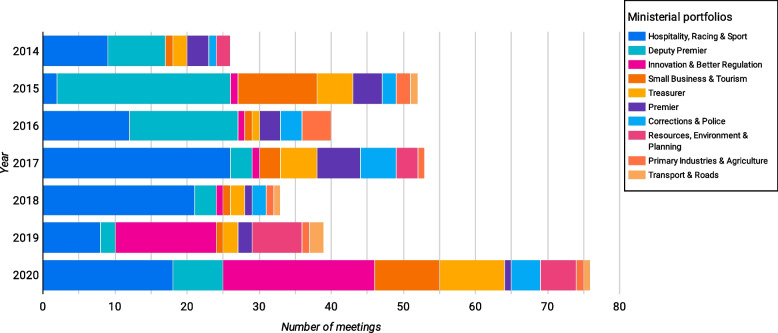
Gambling industry meetings with ministers

Between July 2014 and December 2020, NSW ministers had 20,607 meetings. Of these, 634 meetings were with 'Harmful Industries’: Gambling (*n* = 331), Alcohol (*n* = 158), Ultra-processed foods (*n* = 142) and Tobacco (*n* = 3). Gambling was the most active industry, meeting with ministers almost every month (Table 2, Fig. 2).

**Table 2:**
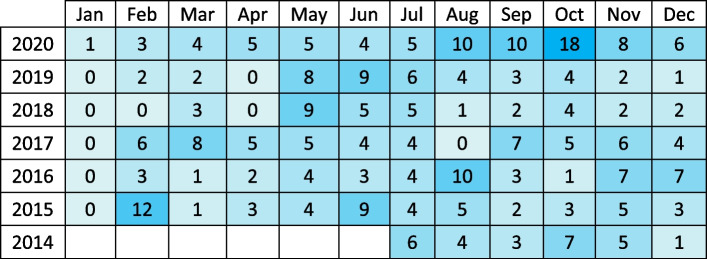
Number of meetings with gambling industry each month

**Fig. 3:**
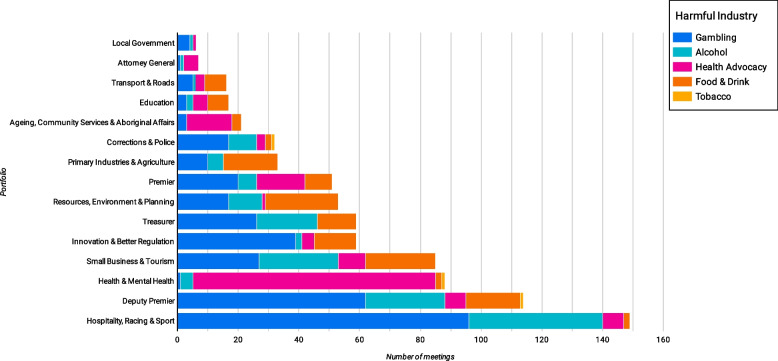
Ministerial lobbying by harmful industries and health advocates

The Hospitality, Racing and Sport portfolio was the portfolio most lobbied by harmful industries (*n* = 149), followed by the Deputy Premier (*n* = 107) (Fig. 3). Most health advocate meetings were with health ministers (*n* = 80) followed by the premier (*n* = 16), and they had no meetings with the treasurer.

**Fig. 4:**
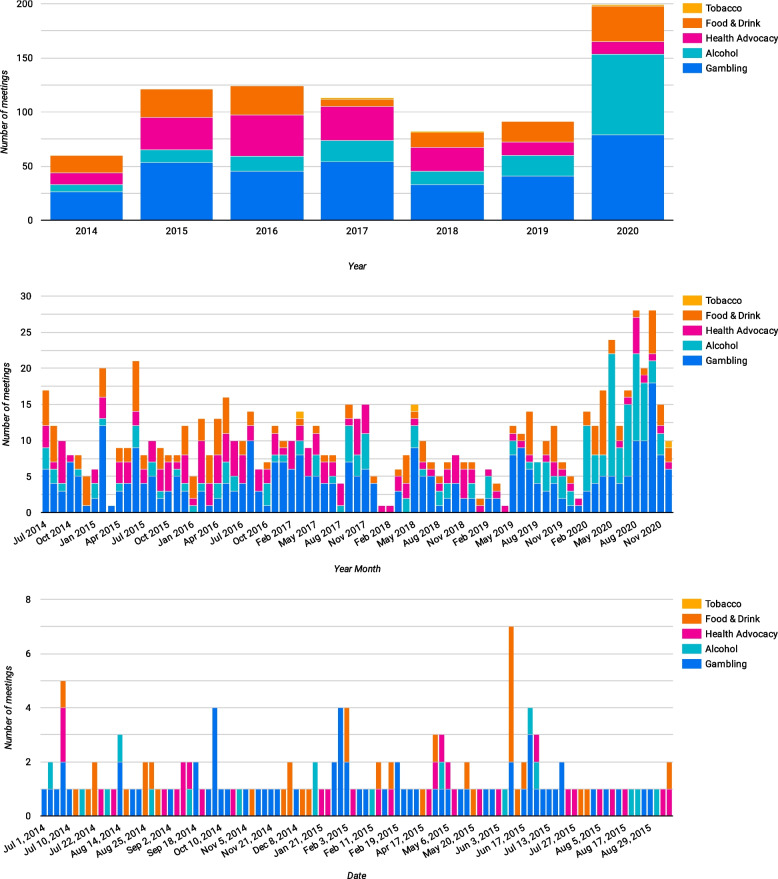
Ministerial lobbying over time (by year, month and day)

Lastly, there was relatively little variation in the number of meetings over time, although 2020 saw a spike in meetings. Looking at meetings over time highlights a key benefit of using Data Studio (or other tools) to visualise the data, as it is possible to drill down to look at meetings by year, month or day, and subsequent analysis could cross reference this to campaign periods or policy debates (Fig. 4).

### Political contributions

**Fig. 5:**
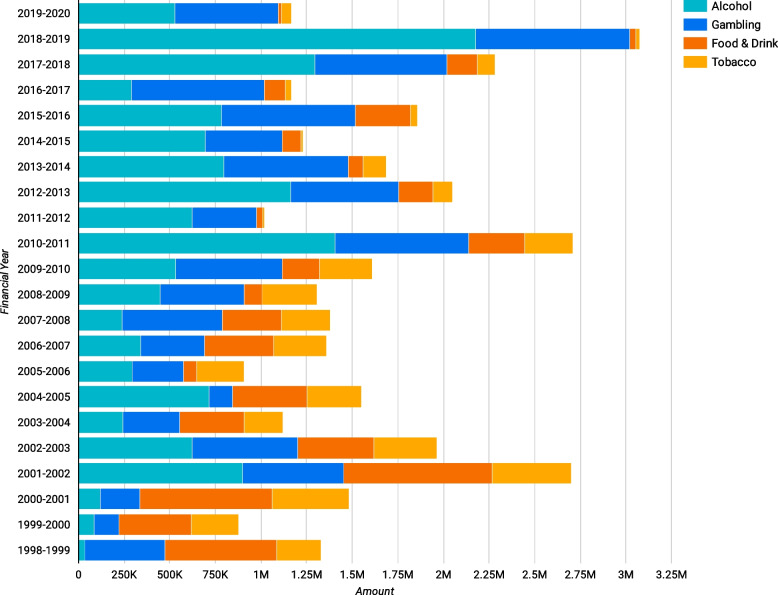
Harmful industry political donations

Between 1998 and 2020, a total of $576,519,472 disclosed donations were made to political parties and other entities. Harmful industries donated $35,823,937: Alcohol ($14,329,566), Gambling ($10,966,200), Ultra-processed foods ($6,144,679) and Tobacco ($4,383,492) (Fig. 5). The Liberal party was the largest recipient of harmful industry donations, with most coming from the alcohol industry followed by gambling, ultra-processed foods and tobacco.

**Fig. 6:**
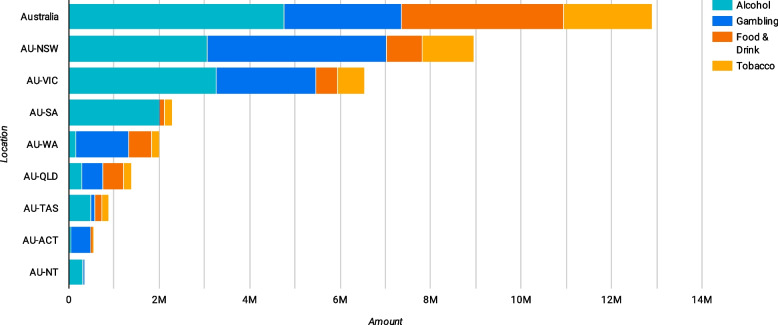
Harmful industry donations by jurisdiction

Most political donations targeted parties at the federal level, though this could also reflect the number of parties without a clear jurisdiction that were similarly coded (Fig. 6).

**Fig. 7:**
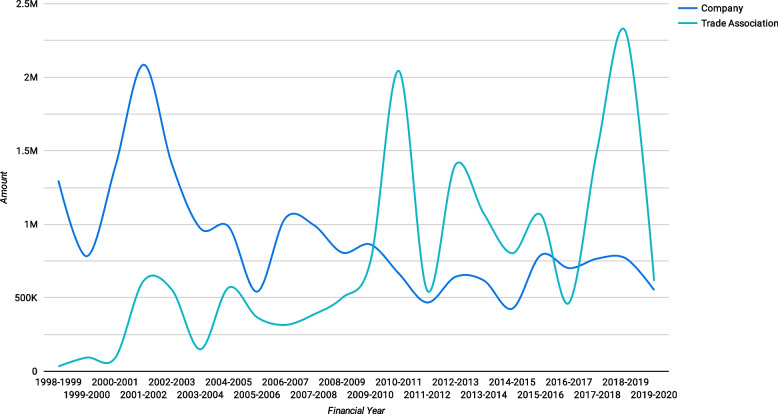
Donations from companies and trade associations

Trade associations played a gradually increasing role, donating slightly more than $33,000 in the 1998–1999 financial year, compared to $2.3 million in 2018–2019 (Fig. 7).

## Discussion

The purpose of this study was to explore the feasibility of monitoring the lobbying and political contribution activities for four ‘harmful industries’ in Australia. For this study, we focused on publicly available data provided by governments, and assessed how easy it was to access, clean, analyse and display in a user-friendly format. Our results illustrate some of the insights that can be found from a preliminary analysis of the data, as well as the many challenges faced in creating a database from incomplete and limited data. Below, we first reflect on some of the empirical findings from our pilot study, and then elaborate on four challenges we faced: 1) variations in the availability of the data, 2) the low level of detail provided, 3) the format of the data, and 4) coding challenges. We also offer suggestions to address these challenges based on our experience and international examples of best practice. We conclude with reflections on how this study could be expanded to other industries or country contexts as well as recommendations for what policy changes would support this work.

Our pilot study provides many interesting insights into the strategies of influence used by commercial organisations. For example, while the gambling industry invested significantly more resources into lobbying than the other sectors examined, the alcohol sector prioritised providing political donations. It is important to note that the lobbying data focused on NSW, where the gambling industry is most active in Australia. Future research that analyses and compares data with other states and territories may identify different patterns depending on the jurisdiction. Interestingly the tobacco industry made minimal representations to ministers in NSW, which is in line with the World Health Organization (WHO) Framework Convention on Tobacco Control. However, the analysis has allowed us to see that the tobacco sector is still providing political donations. The WHO Framework urges member states to either prohibit political donations from tobacco companies or require their full disclosure. This finding of continued political donations, albeit smaller amounts in later years, provides valuable data for tobacco control advocates who are monitoring compliance and the need for improvements within the WHO Framework.

The findings also provide important insights into *who* was being lobbied and *when*. The data demonstrates the deputy premier was the minister receiving the most lobbying from harmful industries, whereas health advocates focussed their efforts on the health minister. Future health advocacy efforts may want to consider targeting similar ministers to those targeted by harmful industries. Undertaking this strategy can ensure both sides of the policy argument are heard by ministers, plus it encourages targeting of more senior ministers within the cabinet who generally have more power and influence in policy making [48]. A significant limitation of the ministerial diaries data is the absence of information about meetings with other public servants, such as senior advisors, who are alternative avenues to influence policy. Research in the EU, for example, highlights the important role of committee chairs and ‘rapporteurs’ in setting policy agendas [49].

The overarching challenge we faced when examining lobbying and political contribution activities was poor data availability. Our initial audit of ministerial diaries, lobbyist registers and political donation records revealed a significant variation in the availability of data to monitor. Currently only three states provide records of ministerial diaries (NSW, QLD, ACT). Lack of responsiveness to timely reporting of political donations and lobbying is unsurprising when only 52% of countries around the world are required to disclose the identity of political donors, further compounding access to this important data [50]. To enable scrutiny of whether and how commercial actors are engaging with politicians requires a far greater level of disclosure. While QLD provides monthly records (published at the end of the following month), both the ACT and NSW provide only quarterly reports resulting in significant delays between when the activity occurs and when information is published. This delay is minimal in comparison with the AEC’s publication of political donations, which can have up to an 18-month gap between the date of a donation and the information release [43]. This challenge is not unique to Australia. Inaccessible data about lobbying and political contributions is the norm internationally [51–53]. Data availability has also presented challenges for public health scholars attempting to monitor the commercial determinants of health, with projects seeking to systematically assess corporate political influence needing to limit the scope of inquiry as data around lobbying was lacking [54].

While there are a myriad of issues associated with reporting of political donations and lobbying, one solution for improving transparency would be to encourage open agendas and real-time reporting. Real-time reporting currently occurs in the state of QLD with political donations, and the authors are not aware of this real-time disclosure occurring elsewhere around the world. However, the use of online spreadsheets and algorithms in Estonia and France have enabled rapid publishing and cross-checking of reporting, both of which could be implemented in Australia [31]. These standards for ensuring that political transparency data is freely available and easily accessible align with the OECD’s push for governments to provide ‘open data’ [55]. Open data refers to the availability, accessibility and reusability of government data. While Australia (along with 78 other countries) participates in the Open Government Partnership, implementation of its national action plan, including actions around political transparency, has been slow [56]. However, with the release of Australia’s draft National Anti-Corruption Commission legislation in late 2022, there are signs of increased political appetite for change [57]. Support for open data policies will not only benefit public health efforts to monitor corporate political activity, but other advocacy efforts likewise concerned with the influence of powerful businesses on political decision making and public integrity.

A second issue is the level of detail provided (or as is more often the case, not provided). A crucial element to understand political activities is the disclosed purpose. While ministerial diaries and some lobbyist records document a purpose, this is often very brief (e.g., ‘Introduction’) and the level of detail varies between states and ministers. Similar challenges around the detail and completeness of disclosed data have been noted in the UK for political donations and lobbying registers [58]. Moreover, many other forms of lobbying and political contributions are not disclosed here, for example: ‘orange card’ lobbyists (those with passes to access parliament house); in-house lobbyists employed by companies or trade associations (not registered lobbying firms); attendance at dinners, lunches, annual general meetings; phone calls and informal discussions; gifts and others [43]. Most importantly meetings with political advisors are not included. These staff are key influencers of decision-makers and an obvious target for lobbyists. Frequently lobbyist will meet with a senior advisor who will then relay this information directly to the Minister often with a personal recommendation [48, 58]. While the lobbyist registers were not the focus of analysis here, their limitations have been discussed elsewhere, with poor disclosure of the revolving door (the movement of people between employment in the public and private sector) highlighted as key risk for conflicts of interest [43].

Lessons can be learned from Canada and Washington State in the USA who are held up as jurisdictions with some of the most comprehensive reporting requirements. Both require in-house and consultant lobbyists to disclose all meetings with a public office holders; this includes politicians, political staff and government employees [59, 60]. Ireland also provides a useful example of more rigorous reporting requirements, which requires a range of different communication methods to be disclosed, including phone calls, emails, meetings and informal communications to be documented [61]. Requirements to disclose the purpose of lobbying are especially important for transparency. This can include the topics discussed in the meeting, specific bills or legislation discussed, and the policy position of client who hired the lobbyist [62].

A third issue is how well the data is organised and structured. To analyse data at scale, it must be structured and machine readable (for example, CSV, XML, JSON). Most Australian data about lobbying and political contributions is available in.pdf form (some of which are scanned images without readable text). While some governments provide downloadable.csv files, these may not contain all the relevant information. NSW, for example, provides.csv files of its political donations, however these do not contain details of specific donations (e.g., date, recipient, amount), which must be viewed individually on the website. Similarly, QLD provides a downloadable.csv file with basic information (donor; recipient; date; amount), however the specific descriptions (e.g., lunch with [named government representative]) can only be viewed online via individual links (*n* = 13,508). While it is possible to clean and organise pdf files into excel tables, this is extremely time-consuming and not realistic to do at scale. For our pilot, data was manually extracted, transformed into tables and cleaned, a process which took several months.

There are notable examples of governments providing open and accessible data such as Germany’s policy to provide free access to machine-readable data, including metadata descriptions for all federal government data [55]. The Australian Research Data Commons promotes ‘FAIR’ data principles (Findable, Accessible, Interoperable and Reusable), which could be used to develop an Australian open data policy [63]. There are opportunities to use algorithms or machine learning tools to scrape websites or analyse large datasets about lobbying [19, 64]. However a simpler, and more far-reaching approach would be to require governments to provide structured (i.e., organised into a predefined format) data to begin with. One of the aims of the pilot project was to explore the possibility of integrating existing datasets (such as those from the published studies identified in our scoping review). To do this efficiently, it would be helpful to develop standard formats for data collection and analysis, such as the ‘ideal’ table model in Fig. 1, which could then be easily incorporated into a master data warehouse. Recent calls from CDoH researchers to develop frameworks and mechanisms to monitor commercial actor practices would benefit from a unified approach to collecting and organising data, as this would facilitate comparisons amongst different country datasets [7, 65].

A final challenge is the complexity of coding. The sheer volume of information presents challenges for manual coding, although there are tools to automate this process to a degree, such as natural language processing or fuzzy matching clustering algorithms, which have been used previously to analyse lobbying databases [66–68]. One of the most basic challenges is the use of different terms or spellings for the same actor. This issue was most prominent for the donor recipients, with more than 100 spelling variations of the Liberal party of NSW alone. The development of standard reporting terms (such as a Data Dictionary) for AEC donor filings, ministerial diaries or other forms of documentation would greatly simplify the current coding challenges. More fundamentally, the commercial actors of interest are themselves complex. To take Australia’s two largest supermarkets, Woolworths and Coles, as an example, both entities are involved in the alcohol, ultra-processed food and tobacco industries, and until recently Woolworths was also involved in the gambling industry through its ownership of Endeavour Group (which owned 286 hotels in Australia) [69]. Similarly, the Australian Hotels Association promotes the interests of both alcohol and gambling [70]. While this level of nuance can be communicated in more qualitative case studies, to develop an agile data warehouse it is necessary to code each actor to just one industry. This has the potential to portray one industry as more active than it actually is or miss the activity of others. A clear explanation of this decision-making process will be necessary for any research translation activities. Furthermore, political portfolios are not static, they change with new governments and even with a new year. These changes present a challenge when coding – do you follow an individual or do you follow a topic? For this analysis we followed a topic over time, however, we encountered numerous examples of topics which were split, reframed or even dissolved over time.

## Conclusions

This pilot study explored the opportunities and challenges for systematically monitoring corporate political activity. The pilot visualisation tool offers an easy-to-use interface, and the preliminary empirical findings can flag areas for further investigation. The pilot study also faced several limitations as detailed in the discussion, and we conclude by highlighting some opportunities to address these limitations and for future research.

A key takeaway from the pilot study is the need for public health researchers and advocates to be strategic in how they access and analyse data about CDoH. Activities that could be undertaken to support the development of a systematic monitoring system include mapping out public datasets that can be scraped and analysed; identifying and piloting tools to scrape, clean and code large (often messy) datasets; developing common coding frameworks/data dictionaries to facilitate collaborations and comparisons amongst studies; and developing well-designed, searchable databases/warehouses for health organisations and NGOs to use. A further possibility to strengthen the pilot visualisation tool would be to incorporate other data such as campaign and policy timelines, which could help to identify important time periods or ministers for attention. Beyond contextualising political practices, this could also help explore changes in lobbying or political donation practices over time or across industries. Similarly, data from business websites could be used to provide further insights about issues of concern to facilitate interpretation of political practices. Linking different datasets also highlights opportunities for cross-country comparisons. Many of the most influential corporate actors in Australia are transnational companies active in many jurisdictions. Comparing their political practices between countries would strengthen the explanatory capacity of the study, though we note that there are challenges with drawing parallels between the political practices of corporations in different countries due to the different political and regulatory systems, ideologies and histories. It would also contribute empirical data towards political science scholarship that analyses the influence of political and institutional contexts on the behaviour of interest groups [49].

For this project, we focussed on four industry sectors, but future work could expand this to include other sectors relevant to health such as mining, pharmaceuticals, accounting/consulting firms, technology, finance and other industries which have faced public health concerns about their political practices [13]. Lobbying firms often work across multiple industries and companies, so expanding the scope of commercial actors under investigation can enable more granular comparisons of patterns of behaviour and determine whether some industry sectors had greater access to political decision makers than others (suggesting undue influence was being exerted). This could also help to inform decisions about what rules might be needed to avoid undue influence and ensure more balanced advocacy opportunities. Additional coding parameters could allow for more flexible or granular analysis, such as the ability to select whether multi-industry actors (e.g., the Australian Hotels Association) code to alcohol or gambling.

Finally, to progress this work and ensure continuity requires sustainable funding (which is increasingly scarce within academia). Thus, a key issue is resourcing, and identifying who could be an appropriate host to fund, build and maintain a tool such as this in Australia or overseas. It is also important to highlight that while the pilot project explores the possibility for researchers to monitor corporate political strategies, such monitoring activities are usually the domain of NGOs and civil society organisations (e.g., Transparency International, Corporate Accountability). Governments must play a key role to ensure that a robust system is in place that provides easily accessible, searchable, and reusable data. New policies and regulations mandating better transparency and disclosure of corporate political activity will be necessary to support public health efforts to monitor the commercial determinants of health. Lessons can be learned internationally about what frameworks and policies support high quality transparency, such as the OECD’s 2020 survey of lobbying regimes [31]. These substantial issues notwithstanding, we are optimistic that this pilot demonstrates a potential case to support the development of mechanisms to monitor commercial political activity, and that this work would contribute to efforts to improve public health, environmental sustainability, human rights and democracy.

## Data Availability

The data analysed in this study is publicly available from the websites provided in the article. The coding frameworks used during the current study are available from the corresponding author on reasonable request.

## Abbreviations

CDoH: Commercial determinants of health
COI: Conflicts of interest
AEC: Australian Electoral Commission
NSW: New South Wales
QLD: Queensland
WHO: World Health Organization
